# Mucin staining is of limited value in addition to basic immunohistochemical analyses in the diagnostics of non-small cell lung cancer

**DOI:** 10.1038/s41598-018-37722-0

**Published:** 2019-02-04

**Authors:** Patrick Micke, Johan Botling, Johanna Sofia Margareta Mattsson, Maria Planck, Lena Tran, Halla Vidarsdottir, Björn Nodin, Karin Jirström, Hans Brunnström

**Affiliations:** 1 Department of Immunology, Genetics and Pathology, Uppsala University, SE-751 85 Uppsala, Sweden; 2 Department of Clinical Sciences Lund, Division of Oncology and Pathology, Lund University, SE-221 00 Lund, Sweden; 3 Department of Respiratory medicine and Allergology, Skåne University Hospital, SE-221 85 Lund, Sweden; 4 Department of Genetics and Pathology, Division of Laboratory Medicine, Region Skåne, SE-221 85 Lund, Sweden; 5 Department of Surgery, Helsingborg Hospital, SE-251 87 Helsingborg, Sweden

## Abstract

Accurate diagnosis of histological type is important for therapy selection in lung cancer. Immunohistochemical (IHC) and histochemical stains are often used to complement morphology for definite diagnosis and are incorporated in the WHO classification. Our main aim was to compare different mucin stains and assess their value in relation to common IHC analyses in lung cancer diagnostics. Using tissue microarrays from 657 surgically treated primary lung cancers, we evaluated the mucin stains periodic acid-Schiff with diastase (PASD), alcian blue–periodic acid-Schiff (ABPAS) and mucicarmine, and compared with the IHC markers p40, p63, cytokeratin 5, thyroid transcription factor 1 (TTF-1), napsin A and cytokeratin 7. Ten or more cytoplasmic mucin inclusions in a tissue microarray core were seen in 51%, 48% and 31% of the 416 adenocarcinomas and 3%, 4% and 0.5% of the 194 squamous cell carcinomas with PASD, ABPAS and mucicarmine, respectively. Diagnostic pitfalls, such as entrapped benign epithelium, apoptotic/necrotic cells and glycogen, partly differed for the mucin stains. TTF-1 and napsin A IHC stainings had similar specificity but better sensitivity for adenocarcinoma than the mucin stains, but addition of PASD or ABPAS identified more tumors as adenocarcinomas (n = 8 and n = 10, respectively) than napsin A (n = 1) in cases with solid growth that were negative for TTF-1 and p40. We conclude that PASD and ABPAS have similar diagnostic performance and that these markers are of value in poorly differentiated cases. However, morphology and TTF-1 and p40 IHC staining is sufficient for correct diagnosis in most non-small cell lung cancers.

## Introduction

Pulmonary non-small cell carcinoma (NSCC) is a heterogeneous group of disorders mainly comprised of adenocarcinomas (AC) and squamous cell carcinomas (SqCC). Distinction of these two entities is of importance for treatment selection as pemetrexed and bevacizumab are used in AC and not in SqCC^1,2^. Furthermore, specific genomic alterations in the EGFR, ALK and ROS1 genes are mainly found in AC^3^, why molecular testing is often only considered for non-squamous NSCC or NSCC with an AC component^4^.

Most patients are diagnosed with advanced disease, why the diagnostic material typically consists of small biopsies or cytology samples, where immunohistochemical (IHC) and histochemical stains are often needed to complement morphology for definite diagnosis. Commonly used IHC markers are p40, p63 and cytokeratin (CK) 5 for SqCC and thyroid transcription factor 1 (TTF-1) and napsin A for AC^5,6^. Traditionally, histochemical staining for mucin is also used for visualization of mucin inclusions for AC diagnosis in morphologically unclear cases^5^. However, the number of routine ancillary stains should be kept to a minimum not to waste tumor material that may be needed for treatment predictive analyses.

There are several different mucin stains^7^. Periodic acid-Schiff with diastase (PASD) for glycogen digestion or alcian blue–periodic acid-Schiff (ABPAS) for staining of additional acid mucins are probably the most commonly used in the diagnostics of lung cancer^8,9^. Mucicarmine is known to have a lower sensitivity for lung AC^7^, but is still used in clinical diagnostics for various purposes and in some lung cancer studies^10^.

A comprehensive evaluation of different mucin stains including a comparison with current IHC markers in lung cancer are to our knowledge missing in the literature. Therefore, the aim of our study was to compare PASD, ABPAS and mucicarmine to assess any differences in staining properties including optimal cutoff levels and investigate their value compared to common IHC stains in the diagnostics of pulmonary NSCC.

## Material and Methods

### Study population

The cases for evaluation included resected primary lung cancers from three different cohorts; the Uppsala 2006–2010 cohort, the Southern Swedish Lung Cancer Study cohort and the Malmö Diet and Cancer cohort^6^. Carcinoid tumors and cases with neoadjuvant treatment were not included in the Uppsala and Southern Sweden cohorts. Otherwise, resected cases were unselectively included in all three cohorts.

Two (Uppsala and Malmö cohorts) or three (Southern Sweden cohort) cores with tumor tissue, 1 mm in diameter, from each case were available on tissue microarrays (TMA). The cases have previously been re-evaluated and examined with various IHC markers and the diagnoses have been updated in adherence with the WHO classification from 2015^5,6^. Briefly, all tumor slides were assessed for morphology, while TMAs were used for IHC staining. In poorly differentiated cases that were negative for e.g. SqCC and AC markers, additional IHC staining and, if needed, mucin staining were performed on whole tumor sections. Neuroendocrine IHC markers were available on whole tumor sections for cases with neuroendocrine morphology and for all cases on TMAs (synaptophysin, CD56, chromogranin A; unpublished data).

All markers relevant for the present study could be evaluated in at least 200 tumor cells in 339, 203 and 115 lung cancers from the Uppsala, Southern Sweden and Malmö cohorts, respectively, and these cases were included in the analysis. Twelve cases were included in both the Southern Sweden and Malmö cohorts, and these were only included once each in the present study. In 41 cases from the Uppsala cohort (32 AC and 9 SqCC), a single core from a matched metastasis (mainly lymph node or brain metastasis or local recurrence) was evaluable for mucin staining in the TMAs.

In addition, a cohort of consecutive resected epithelial metastases to the lungs, surgically treated at the Skåne University Hospital in Lund in 2000–2014, was investigated (ABPAS only)^11,12^. In total, 419 pulmonary metastases from 334 patients were evaluable on TMA slides with two cores, 1 mm in diameter, for each case.

### Staining

Consecutive 4 micrometer thick TMA sections were stained with PASD, ABPAS (pH 2.5 for alcian blue) and mucicarmine at the Department of Genetics and Pathology, Lund, Sweden. For mucicarmine, small intestine was used as control tissue. For PASD and ABPAS, small intestine and liver was used as control tissue but only for each run, not on each slide. IHC stained sections for p40, p63, CK5, TTF-1 and napsin A were available from previous investigations (these were not consecutive to the sections for mucin stains, but there was no significant difference in morphological appearance of the tumors between the sections)^6^. The antibody clones and concentrations were p40 clone BC28 1:50 (Biocare Medical, Concord, CA), p63 clone 4A4 ready-to-use, TTF-1 clone 8G7G3/1 ready-to-use (both Ventana Medical Systems, Tucson, AZ), CK5 clone XM26 1:25, TTF-1 clone SPT24 1:100, and napsin A clone IP64 1:20 (all three Leica Biosystems, Nussloch, Germany). Control tissue was used on each slide. As part of the present study, IHC staining for CK7 was also performed with clone SP52 ready-to-use (Ventana) with liver as control tissue.

### Evaluation

All slides were evaluated by a pathologist working with thoracic pathology on a daily basis (H.B.). The sections stained for mucin were all evaluated at 400x magnification. During the evaluation, the investigator was blinded to the results of the other mucin stains and of the IHC staining. The investigator was also blinded to the diagnoses, though in most cases the diagnosis was evident based on morphology of the tumor tissue on the TMA sections. Cases with a discrepant result between mucin stains were reviewed again for further comparison.

For each case, the number of cells with cytoplasmic mucin inclusions in the TMA core with the most inclusions were counted and registered on a scale 0–10, equal to the absolute number of inclusions except that 10 also included cases with more than 10 inclusions. In addition, presence of extracellular mucin was noted in cases with no cytoplasmic inclusions. Presence of only cytoplasmic globules that were magenta-colored in ABPAS (hyaline/eosinophilic intracytoplasmic globules)^13^ was also noted. For AC cases, it was noted if there was only solid growth with no glandular formations in the TMA sections, and if glycogen was visible in the ABPAS staining. For pulmonary mucinous AC and metastases to the lungs, the presence of acid mucins was noted for APBAS. Especial care was taken not to interpret apoptotic or necrotic cells, macrophages, trapped benign epithelium, glycogen or stromal mucin as cytoplasmic mucin.

For IHC staining, the fraction of positive viable tumor cells in the TMA cores was divided into five categories for each case: less than 1%, 1–9%, 10–24%, 25–49%, and 50% or more.

### Statistical analysis

Both Wilcoxon signed-rank test and paired Student’s t-test, or both one-way analysis of variance (ANOVA) and Kruskal-Wallis test when appropriate, were used to compare the number of cytoplasmic inclusions between the different mucin stains and between different groups of relevance. A p-value of < 0.05 was considered statistically significant. Receiver operating characteristic (ROC) curve analysis was used to compare the diagnostic value of different mucin and IHC stains. All calculations were performed using MedCalc Statistical Software version 14.10.2 (MedCalc Software BVBA, Ostend, Belgium).

### Ethics

The analyses of the material were conducted in adherence with the Declaration of Helsinki (incl. informed consent) and have been approved by the regional ethical review boards in Uppsala (Dnr 2012/532) and Lund (Dnr 2007/445, 2008/35 and 2014/748, and Dnr 2004/762 and 2008/702, respectively).

## Results

The complete diagnostic material from 645 resected primary lung cancers was investigated and representative tumor areas were available on TMAs. This included tissue cores from 402 AC, 188 SqCC, 8 adenosquamous carcinomas, 8 large cell carcinomas (LCC), 6 sarcomatoid carcinomas, 23 large cell neuroendocrine carcinomas (LCNEC), 3 small cell carcinomas, and 7 carcinoid tumors.

The AC component of all 8 adenosquamous carcinomas and 6 of the 8 combined LCNEC cases (all with an AC component) were evaluable and these were grouped with the AC cases as separate tumors, leading to 416 AC cases. Similarly, the SqCC component from 6 of the 8 adenosquamous carcinomas were evaluable and these were grouped with the other SqCC for a total of 194 cases. Consequently, the total number of analyzed cases was 657, see Table 1.

**Table 1:** Frequency of primary lung cancer cases with 1+/10+ cytoplasmic mucin inclusions in a tissue microarray core and range of number of mucin inclusions (in parenthesis).

Diagnosis	No. of cases	PASD	ABPAS	Mucicarmine
Adenocarcinoma	416	81%/51% (0–10+)	78%/48% (0–10+)	58%/31% (0–10+)
Squamous cell carcinoma	194	11%/2.6% (0–10+)	9.8%/4.1% (0–10+)	4.6%/0.5% (0–10+)
Large cell carcinoma	8	38%/0% (0–5)	38%/0% (0–6)	13%/0% (0–1)
Sarcomatoid carcinoma	6	50%/50% (0–10+)	50%/50% (0–10+)	50%/33% (0–10+)
Small cell carcinoma	3	0%/0% (0)	0%/0% (0)	0%/0% (0)
Large cell neuroendocrine carcinoma	23	4.3%/0% (0–1)	0%/0% (0)	0%/0% (0)
Carcinoid tumor	7	0%/0% (0)	0%/0% (0)	0%/0% (0)
All	657	56%/34% (0–10+)	53%/32% (0–10+)	39%/20% (0–10+)

Abbreviations: ABPAS, Alcian blue–periodic acid-Schiff; PASD, Periodic acid-Schiff with diastase.

The LCC and sarcomatoid carcinomas (whereof five were pleomorphic carcinomas with an AC component and one was a giant cell carcinoma) were grouped with the AC cases in some of the analyses as the handling and treatment is typically the same for these groups (and different compared to SqCC and neuroendocrine tumors).

### Mucin staining

**Figure 1:**
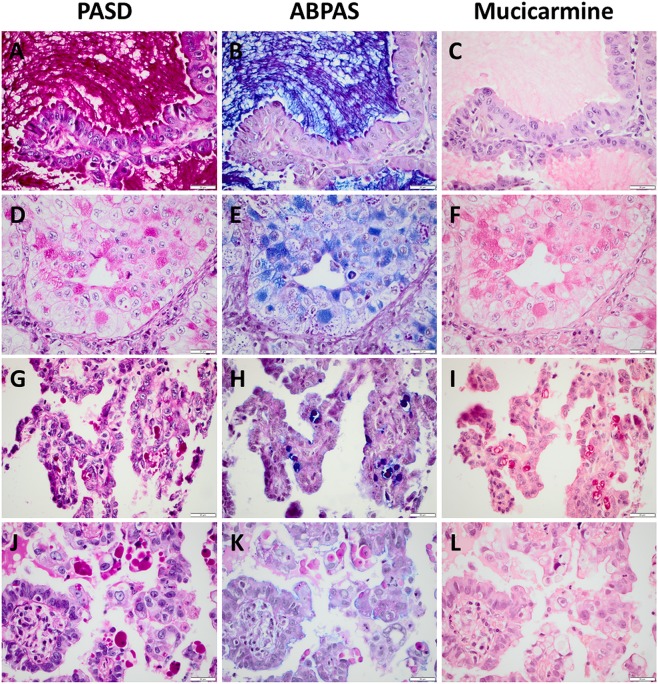
Staining with PASD, ABPAS and mucicarmine in four pulmonary adenocarcinomas stained. (**A**–**C**) A case with cytoplasmic and abundant extracellular mucin. (**D**–**F)** A case with only blue-colored mucin and visible glycogen in ABPAS. (**G**–**I)** A case with blue and magenta-colored mucin in ABPAS. (**J**–**L**) A case with cytoplasmic globules, magenta-colored in ABPAS and PASD. Scale bar is 20 micrometer.

Cytoplasmic and extracellular mucin was magenta-colored in PASD staining, blue to purple (i.e. positive for alcian blue/acid mucins) and/or magenta-colored (i.e. positive for PAS/neutral mucins) in ABPAS and red in mucicarmine. Examples of the three mucin stains in representative AC cases are seen in Fig. 1 for visualization of differences in color and staining properties.

**Figure 2:**
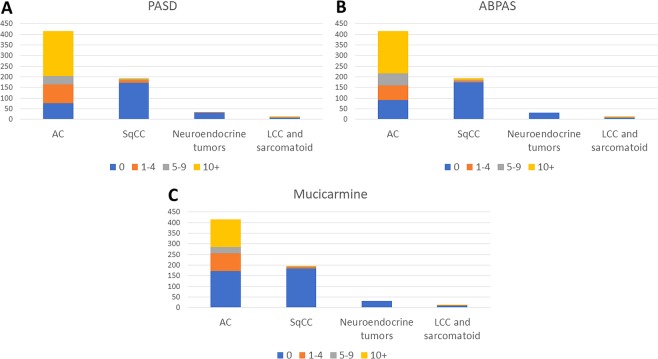
Distribution of number of cytoplasmic mucin inclusions, based on the TMA core with the most inclusions, in 657 lung cancers stained with PASD, ABPAS and mucicarmine.

The frequency of cases with 1+ and 10+ (the latter practically correlating to the number of inclusions defining AC according to the WHO classification)^5^ cytoplasmic mucin inclusions, based on the TMA core with the most inclusions for each case, is found in Table 1 and also visualized in Fig. 2.

Presence of only cytoplasmic globules were seen in 23 (6%) of the AC cases, whereof 19 with 10+ globules in PASD and/or ABPAS, and in 2 (1%) of the SqCC cases (included as cytoplasmic inclusions in the numbers above). The globules were magenta-colored in PASD and ABPAS, while being very pale red in mucicarmine and typically not stronger than the background. See Fig. 1J–L for an example.

There were 10 AC cases with a presence of limited but clearly visible extracellular mucin but without cytoplasmic mucin in any of the three stainings. All cases were non-mucinous and exhibited lepidic and/or acinary growth pattern. Extracellular mucin was also seen in one SqCC and four LCNEC cases that was not obvious stromal mucin or from trapped benign epithelium.

### Challenges to correctly interpret mucin stainings

**Figure 3:**
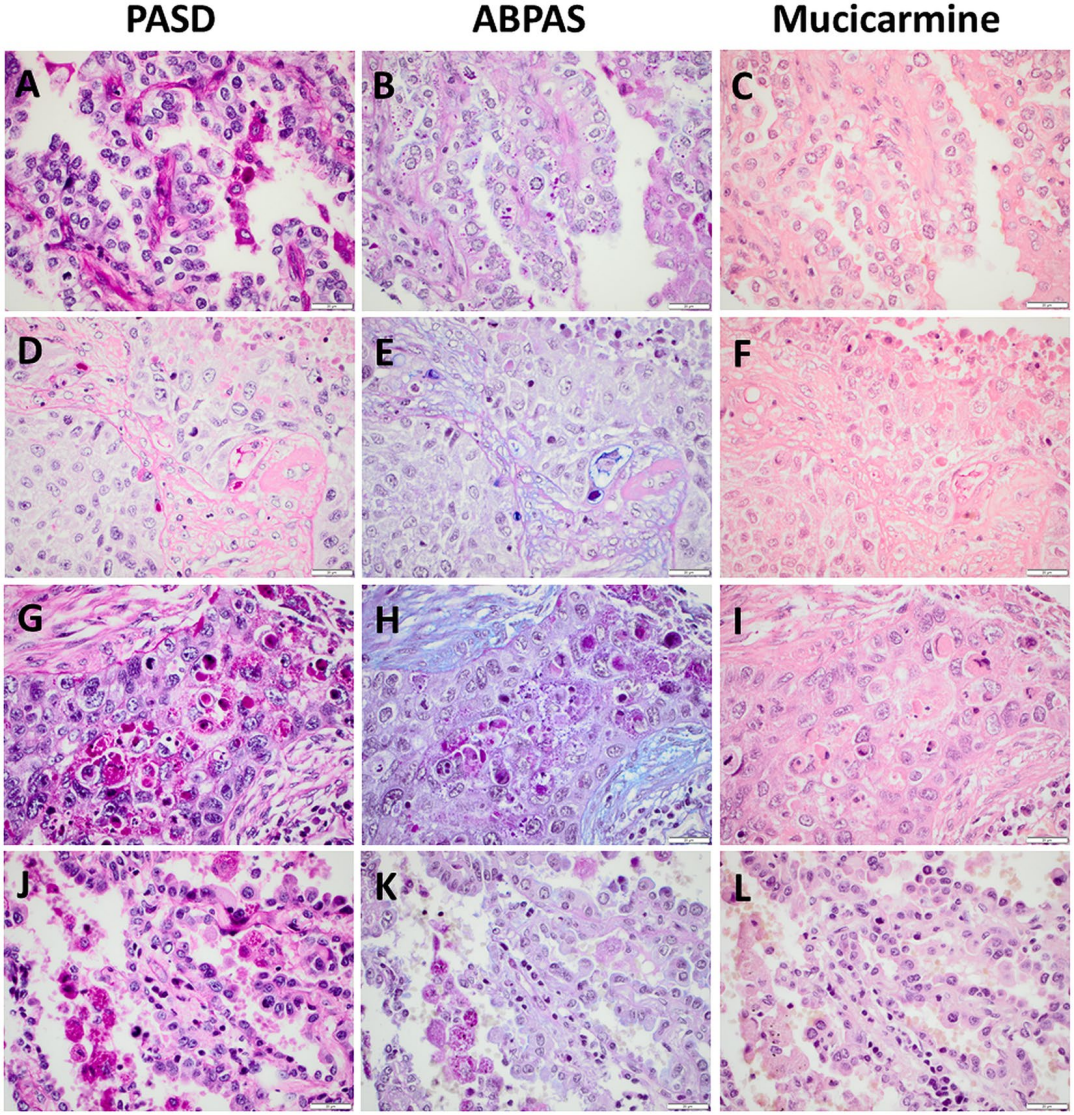
Examples of diagnostic pitfalls in evaluation of PASD, ABPAS and mucicarmine in four pulmonary carcinomas. (**A**–**C)** A case of adenocarcinoma where it was difficult to distinguish glycogen from cytoplasmic globules (and some adjacent macrophages). (**D**–**F)** A case of squamous cell carcinoma with mucin in trapped benign epithelium (also positive for e.g. TTF-1, not shown). (**G–I**) A case of squamous cell carcinoma with necrosis and stromal mucin. (**J**–**L**) A case of adenocarcinoma with adjacent macrophages. Scale bar is 20 micrometer.

Small granules consistent with glycogen was visible in ABPAS staining in 56 (13%) of the AC cases, whereof 16 exhibited only solid growth in the TMA sections (glycogen was more common in SqCC, data not compiled). In contrast with the typical blue to purple mucin inclusions and the magenta-colored cytoplasmic globules, glycogen was typically very small granules with a darker magenta color. See Fig. 1D–F for an example. However, in odd cases it was difficult to distinguish small magenta-colored cytoplasmic globules visible in PASD and ABPAS from glycogen in the latter staining. See Fig. 3A–C for an example.

**Figure 4:**
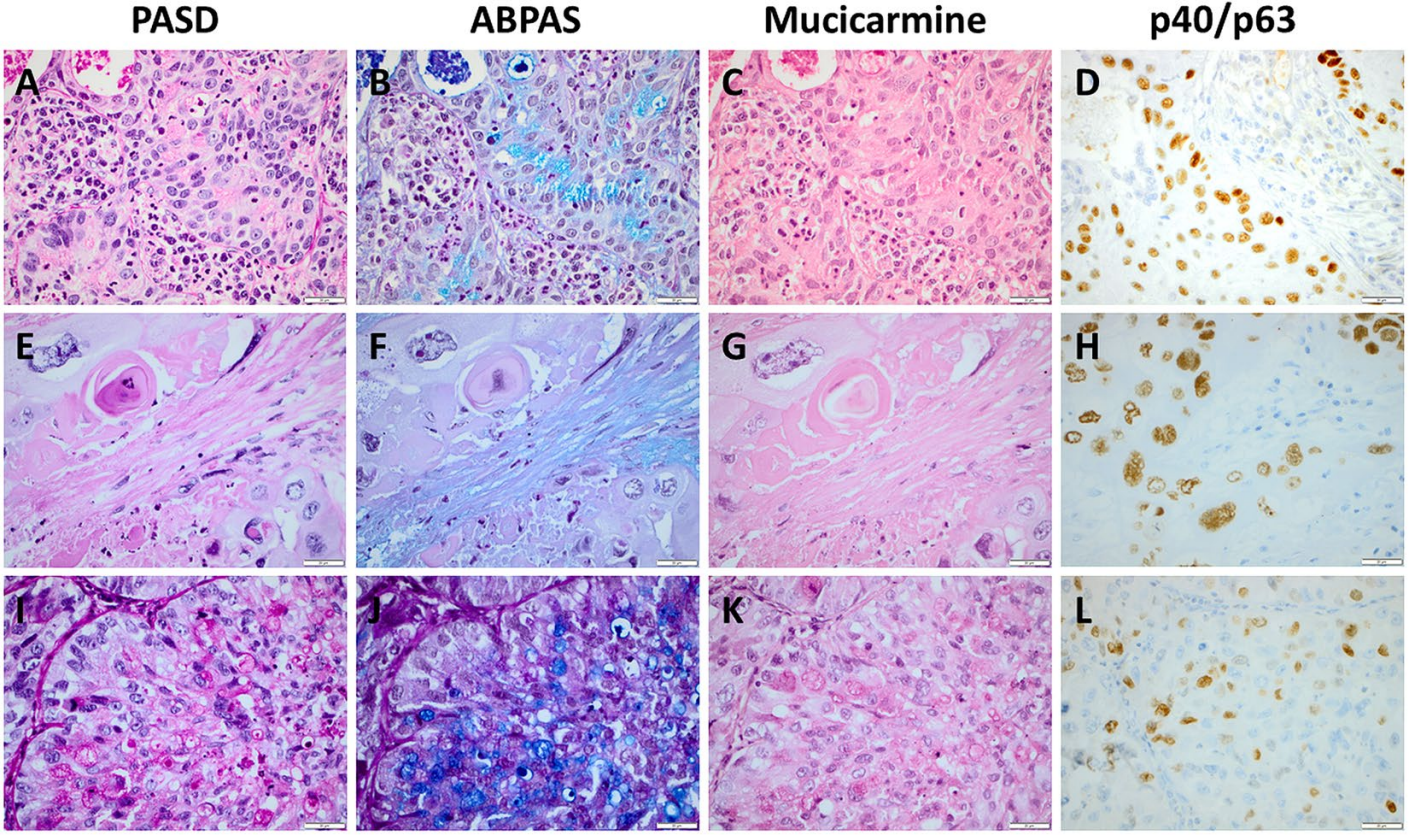
Staining with PASD, ABPAS, mucicarmine and p40 (**D,L**) or p63 (**H**) in three pulmonary carcinomas. (**A**–**D**) A case of squamous cell carcinoma with cytoplasmic mucin (and trapped benign epithelium). (**E**–**H**) A case of squamous cell carcinoma with keratinization (and necrosis and stromal mucin). (**I**–**L**) A case of adenocarcinoma previously diagnosed as squamous cell carcinoma based on squamoid appearance and focal positivity for p63 (p40 and CK5 were negative, not shown). Scale bar is 20 micrometer.

Apoptotic/necrotic tumor cells regardless of histological type were typically magenta-colored in PASD and ABPAS and slightly more red than the reddish background in mucicarmine. See Figs 3G–I and 4E–H. Apoptotic/necrotic cells were occasionally a cause for care especially in PASD, while keratinizing tumor cells (see Fig. 4E–H) did not pose any significant diagnostic problems.

Among benign cells, a diffuse or granular cytoplasmic magenta staining of macrophages could be seen in PASD and ABPAS, while in these cases there was only a slight red accentuation compared to reddish background in mucicarmine. See Fig. 3A–C,J–L. Both tumor-infiltrating macrophages (especially in PASD) and trapped benign/reactive alveolar epithelium (see Fig. 3D–F) were occasionally a cause for care. In contrast, mucin in entrapped benign bronchiolar epithelium and reactive type II pneumocytes near a tumor (inconsistently stained) did not cause any diagnostic problems, as did not stromal mucin (see Figs 3G–I and 4E–H).

### Difference in staining properties

Based on the TMA core with the most cytoplasmic mucin inclusions for each of the 657 cases, the mean ± SD number of inclusions was 4.1 ± 4.5 for both PASD and ABPAS, while the median was 2 for PASD and 1 for ABPAS. Both paired Student’s t-test and Wilcoxon signed-rank test were non-significant (p = 0.53 and p = 0.71, respectively). The mean ± SD and median number of inclusions for mucicarmine were 2.6 ± 4.0 and 0, respectively, and the numbers were significantly lower than for both ABPAS and PASD in pairwise analyses with both paired Student’s t-test and Wilcoxon signed-rank test (all p < 0.0001).

More AC cases were positive for PASD and ABPAS than mucicarmine, see Table 1 and Fig. 2, also true when the 71 AC cases with solid growth pattern in the TMA sections were evaluated. Positivity with 1+ and 10+ inclusions were seen in 54 (76% of 71) and 35 (49%) solid AC cases with PASD, in 47 (66%) and 34 (48%) cases with ABPAS, and in 37 (53% of 70) and 18 (26%) cases with mucicarmine, respectively.

There was a substantial overlap between the mucin stains. In 311 (75%) and 178 (43%) of the 416 AC cases, 1+ and 10+ cytoplasmic mucin inclusions, respectively, were seen with both PASD and ABPAS. In 42 (10% of 416) and 49 (12%) additional AC cases only one of these two mucin stains showed 1+ and 10+ inclusions, respectively. There was only a single case with 10+ cytoplasmic inclusions in mucicarmine that exhibited fewer than 10 inclusions in both PASD and ABPAS.

### Mucin in non-AC cases

The frequency of cytoplasmic mucin inclusions for non-AC cases are seen in Table 1 and Fig. 2. At least one inclusion was seen with any of the three mucin stains in 26 (13%) of the 194 SqCC cases. All these cases were diffusely positive for p40 and/or CK5 (22 cases were positive for both markers). In 3 and 7 SqCC cases, 10+ inclusions were seen with both PASD and ABPAS and with just one of these two stains, respectively.

An example of SqCC with mucin inclusions is seen in Fig. 4A–D, while Fig. 4E–H shows the appearance of keratinization in the different mucin stains. A few of the SqCC cases with cytoplasmic mucin inclusions had focally cylindrical tumor cells with mucin, which may represent mixed SqCC and AC but with too limited AC component for the case to be classified as adenosquamous carcinoma (the diagnoses were based on whole tumor sections). None was consistent with mucoepidermoid carcinoma.

TTF-1 clone SPT24 and CK7 were positive in one (4%) and 16 (62%) of the 26 SqCC cases with any mucin positivity, respectively, compared to 5 (3%) and 83 (43%) of all 194 included SqCC cases. One other case exhibited focal positivity for napsin A (<10% of the cells) while TTF-1 clone 8G7G3/1 was negative in all cases. From previous investigations, data from targeted next-generation sequencing (NGS) was available for 16 of the 26 cases. No pathogenic *EGFR* mutations were found, while a *KRAS* mutation was seen in one case. This was a poorly differentiated cancer positive for CK5 and CK7 and negative for p40, p63 and AC markers, while 5 mucin inclusions were seen with both PASD and ABPAS (none with mucicarmine). In one and four of the 16 tested cases, a *BRAF* and *PIK3CA* mutation was found, respectively. Both FISH and IHC were negative for *ALK* in all 12 tested cases.

One case with squamoid morphology, positive CK7 (all cells) and p63 (about 10% of the cells) and negative p40, CK5, TTF-1 and napsin A had previously been classified as non-keratinizing SqCC without mucin staining. Here, 10+ cytoplasmic mucin inclusions were seen with all three mucin stainings, and the widespread mucin inclusions were judged to trump p63. The case was therefore reclassified as AC with solid growth and included in the AC group above and in all analyses of the present study. See Fig. 4I–L for this case.

The overlap between PASD and ABPAS was perfect for the three sarcomatoid carcinomas (all pleomorphic carcinomas with an AC component) and the three LCC with cytoplasmic mucin. Mucicarmine stained the same cases when positive. The single case of LCNEC with a single mucin inclusion in PASD was a combined LCNEC with an AC component where the mucin inclusion was found in the neuroendocrine component (the AC component was not present in the TMA).

There was one LCC where an old mucin stain (from the clinical situation) on a whole tumor section had not revealed enough mucin inclusions for AC diagnosis. Now we found numerous (10+) inclusions with PASD and ABPAS on the TMA section. This case was reclassified as AC with solid growth and included in the AC group above and in all further analyses.

### Comparison with IHC staining

To evaluate the diagnostic value of the mucin stains in comparison with commonly used IHC stainings, ROC curve analyses for separation of AC, LCC and sarcomatoid carcinomas from SqCC and neuroendocrine tumors were performed and the results for the optimal cutoffs are seen in Table 2. For all three mucin stains, 1+ cytoplasmic inclusions was the best cutoff in the ROC curve analyses, while 10+ inclusions correspond with recommendations^5^.

**Table 2:** Optimal cutoffs (1+ cytoplasmic inclusions for the mucin stains) and the sensitivity and specificity for different mucin stains and immunohistochemical markers to separate adenocarcinomas, large cell carcinomas and sarcomatoid carcinomas from squamous cell carcinomas and neuroendocrine tumors based on 657 primary lung cancers.

Staining	Cutoff	Sensitivity (%)	Specificity (%)	AUC
PASD	1+ inclusions	80	90	0.87
	10+ inclusions	50	98	0.74
ABPAS	1+ inclusions	77	92	0.85
	10+ inclusions	47	96	0.72
Mucicarmine	1+ inclusions	58	96	0.78
	10+ inclusions	31	100	0.65
TTF-1 clone SPT24	≥10%	88	87	0.89
TTF-1 clone 8G7G3/1	≥1%	86	90	0.89
Napsin A	≥1%	86	98	0.93
CK7	≥50%	96	65	0.81
p40	<10%	100	81	0.91
p63	<50%	96	82	0.88
CK5	<1%	99	84	0.92

Abbreviations: ABPAS, Alcian blue–periodic acid-Schiff; AUC, area under curve; CK, cytokeratin; PASD, Periodic acid-Schiff with diastase; TTF-1, thyroid transcription factor 1.

Exclusion of the neuroendocrine tumors in the calculations (to essentially investigate the separation of AC from SqCC) did not significantly affect the sensitivity and specificity for the mucin stains, napsin A and CK7 (0–2 percentage points difference compared to the numbers in Table 2). However, the specificity was increased considerably for TTF-1 (both clones), p40, p63 and CK5 if the neuroendocrine tumors were excluded in the calculations. Using 10% or 25% positive tumor cells as cutoffs for p40 or CK5 did not significantly affect the sensitivity and specificity (0–2 percentage points difference compared to the numbers in Table 2).

### Analysis of diagnostic panels

Based on the findings in the TMA sections, expression of p40 in ≥10% of the tumor cells and/or presence of obvious keratinization identified 184 (95%) of 194 SqCC (among non-SqCC cases, one AC with solid growth was positive for p40 but also for both TTF-1 clones in >50% of the cells). Correspondingly, a positive TTF-1 (clone SPT24) in ≥10% of the tumor cells and/or presence of obvious glands in the TMA sections identified 404 (97%) of 416 AC but also included 25 of 33 neuroendocrine tumors, 6 of 194 SqCC (all positive for CK5 and p40 and negative for TTF-1 clone 8G7G3/1 and napsin A) and three sarcomatoid carcinomas.

The 41 cases (6% of 657) with negative p40 and TTF-1 and no obvious keratinization or glands consisted of 12 AC, 10 SqCC, 8 LCC, 8 neuroendocrine tumors and 3 sarcomatoid carcinomas. Addition of napsin A, regardless of cutoff 1% or 10%, identified one additional AC (negative for mucin), while addition of a mucin stain with 10+ inclusions as cutoff identified 8 and 10 additional AC cases with PASD and ABPAS, respectively, and stained one additional pleomorphic carcinoma. Addition of CK5 with cutoff 10% positive tumor cells for a positive staining identified an additional 8 SqCC cases (and one additional AC with solid growth was positive for CK5 but also for both TTF-1 clones in >50% of the cells).

**Figure 5:**
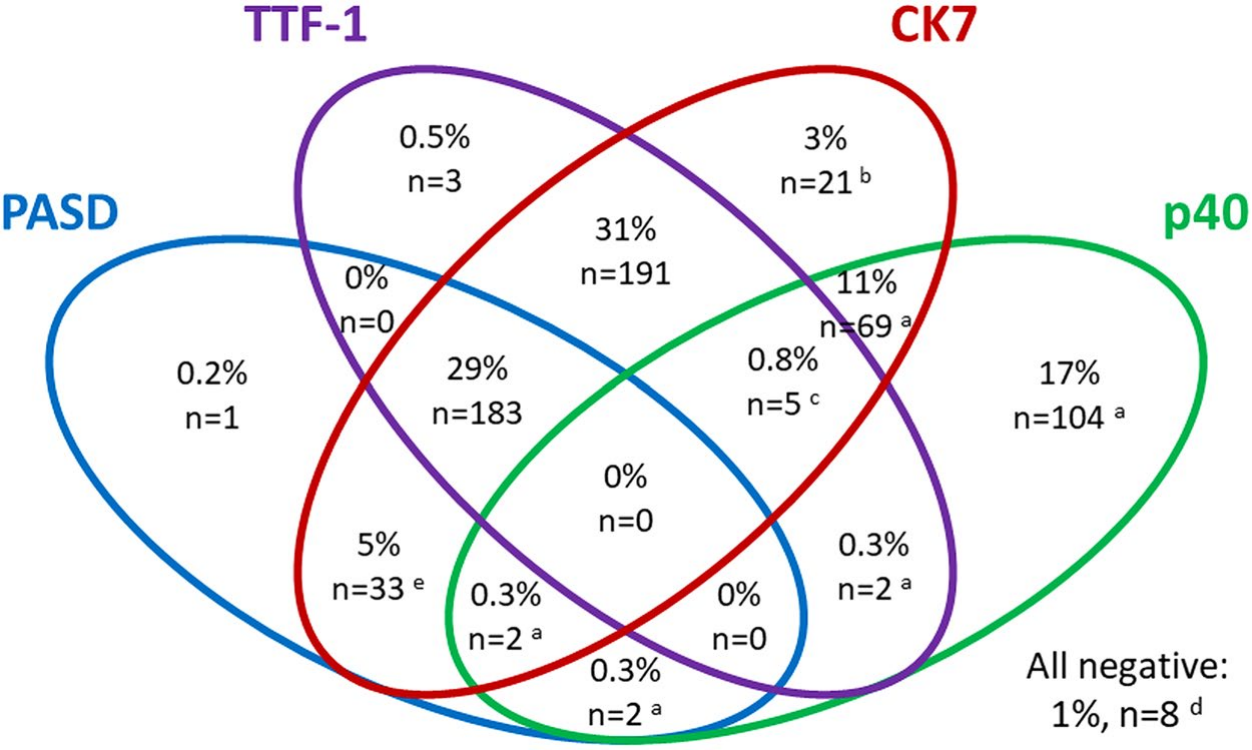
Staining profiles of 624 pulmonary NSCC cases (neuroendocrine tumors excluded) with 10+ cytoplasmic inclusions for PASD and ≥10% positive tumor cells for the IHC stains p40, TTF-1 (clone SPT24) and CK7 as cutoff for a positive marker. The number of SqCC cases is indicated: a, all; b, 7; c, 4; d, 3; e, 1 SqCC.

Among the 41 cases with negative p40 and TTF-1 and no obvious keratinization or glands, CK7 was positive (≥50% of the cells in all cases with any positivity) in 10 of the 12 AC, 7 of the 10 SqCC, 5 of the 8 LCC, 2 of the 8 neuroendocrine tumors and 2 of the 3 sarcomatoid carcinomas. In all, CK7 was positive in ≥50% of the cells in 494 (75%) of all 657 investigated lung cancer cases, and in 405 (97%) of 417 AC and 64 (33%) of 194 SqCC. In Fig. 5, the relation between p40, TTF-1, PASD and CK7 is shown for the 624 NSCC cases in the study population (the 33 neuroendocrine tumors excluded).

### Mucin staining in relation to smoking and neuroendocrine expression

The mean number of mucin inclusions in AC cases was marginally higher for current smokers than former smokers (>12 months since cessation) and for former smokers than never-smokers in all mucin stains (e.g. mean score 6.6 vs. 5.9 vs. 5.6 for PASD), but the differences were non-significant using both ANOVA and Kruskal-Wallis test (all p > 0.2). Less differences and no trends were seen if all histological types were included in the analyses. Also, there were no significant differences in number of mucin inclusions in AC cases with expression of any neuroendocrine marker (≥10% of the cells) compared to other AC (all p > 0.3).

### Mucin staining in metastases of lung cancer

A matched metastasis was evaluable for the mucin stains in 32 AC and 9 SqCC. The mean number of mucin inclusions were marginally higher in the primary tumors for all mucin stain (e.g. mean score 4.9 vs. 4.2 for PASD) but not significantly using Wilcoxon signed-rank test or paired Student’s t-test (all p > 0.2). Using 10+ inclusions as cutoff, there was a difference between the primary tumor and metastasis in 11 (27%), 7 (17%) and 6 (15%) of these 41 cases for PASD, ABPAS and mucicarmine, respectively (in six cases more mucin inclusions were seen in the primary tumor and in five cases more were seen in the metastasis).

### Alcian blue staining in pulmonary mucinous AC and metastases to the lungs

All 21 pulmonary mucinous AC included in the cohorts exhibited blue cytoplasmic inclusions, i.e. positive for alcian blue/acid mucins, while three of these also had magenta-colored inclusions, i.e. also positive for PAS/neutral mucins. In total, 110 of 419 metastases to the lungs had 10+ mucin inclusions in ABPAS staining, and the inclusions were positive for alcian blue/acid mucins in 101 of these cases. These metastases included 80 of 274 colorectal AC, 5 of 26 breast AC (whereof one mucinous AC), 5 of 5 pancreatic AC, 4 of 17 non-squamous gynecological cancers (whereof two mucinous AC), 4 of 4 appendiceal AC, 1 of 11 SqCC (the positive case was of esophageal origin), 1 of 2 small intestine AC, and 1 of 1 gall bladder AC while the criteria was not fulfilled by any of 40 renal AC, 11 prostatic AC, 8 urothelial carcinomas, 6 adenoidcystic carcinomas, 5 thymomas, 4 liver cell carcinomas, 3 thyroid AC, 1 esophageal AC, and 1 basal cell carcinoma.

## Discussion

Our study showed that the mucin stains PASD and ABPAS exhibit high specificity but low sensitivity for AC when the cutoff for positivity was defined as 10+ cytoplasmic mucin inclusions in a 1 mm TMA core (comparable to the WHO recommendation)^5^. The sensitivity increased considerably when the cutoff was lowered to 1+ inclusions. The performance of PASD and ABPAS was generally comparable, while mucicarmine had a limited sensitivity for AC in our material, also supported by the literature^7,10^, indicating that this staining is of low diagnostic value and should not be used in the diagnostics of lung cancer.

Evaluation on TMA sections allows for comparison of many cases and is excellent for comparison of staining properties of similar types of stains if consecutive sections are used. However, our results cannot be fully applied to resected cases, and a higher frequency of mucin positivity has been reported based on whole tumor sections^7,14^. Also, in a previous study of ours including the same cases as in the present study, a positive mucin stain on a whole tumor section (performed only in unclear cases) determined the AC diagnosis in only three cases (0.7%), while glandular formations and/or a positive IHC marker (TTF-1 and/or napsin A) was sufficient for diagnosis for all other AC cases^6^.

Although the comparability of TMAs to resected specimens is clearly limited, the size and tissue content may more resemble biopsies, which is the common diagnostic material in lung cancer patients. In two limited series of small specimens with lung cancer, adding a mucin stain increased the sensitivity for AC from 54% (with only TTF-1) to 69% in the study by Loo and co-workers while it did not add any diagnostic value in the study by Nicholson and co-workers^8,9^. As morphology is maybe better preserved in TMA sections than in small specimens from the clinical setting, the diagnostic value of mucin staining may be underestimated in our material (if disregarding morphology, sensitivity for AC increased from 90% with only TTF-1 to 99% if adding a mucin stain).

Still, our study contributes to identification of practical panels of ancillary stains in the diagnostics of pulmonary NSCC. P40 and TTF-1 (often clone SPT24) is a commonly used panel for pulmonary NSCC when morphology is not perfectly clear, also supported by the guidelines in the WHO classification^5^. This limited panel solves most cases together with morphology and compared to mucin stains evaluation of these IHC markers is somewhat faster and afflicted with fewer diagnostic pitfalls in our experience. However, our data support that addition of a mucin stain in ambiguous cases is sometimes helpful to determine an AC diagnosis.

For instance, if both p40 and TTF-1 are negative, and there are no clear glandular structures or keratinization, addition of CK5 and a mucin stain seems to be a good choice, while napsin A is of limited value. Also, a broad cytokeratin should be considered to confirm epithelial type of malignancy. If CK5 and mucin staining are also negative, the case will often be LCC or a neuroendocrine tumor, while of course metastases to the lungs and, especially if negative broad cytokeratin, non-epithelial tumors must be considered (not investigated in the current study). Based on our and other data, CK7 is neither sensitive for pulmonary NSCC, nor specific for AC^15–18^.

Our data support that presence of mucin inclusions does not exclude a SqCC diagnosis, and although somewhat linked to CK7 expression, mucin does not predict a value of molecular testing for treatable targets in SqCC. Also, we wanted to test the hypothesis that ABPAS may distinguish primary pulmonary mucinous AC from metastases to the lungs, as a limited occurrence of acid mucins has been demonstrated in e.g. gastric cancer^19^. Although there were no metastases to the lungs of gastric origin in our material, our data show that acid mucins are present in metastases of various origin, also previously known for e.g. colorectal cancer^20^.

A point of controversy is if only cytoplasmic mucin should be taken into account^8^, or if also mucin in neoplastic glandular acini should be considered as proof of AC differentiation^7^. We focused only on cytoplasmic mucin but agree that staining of extracellular mucin can be worthful, e.g. to highlight gland formations if rare. We also identified the cases with only cytoplasmic globules that were magenta-colored in ABPAS (and PASD) and chose to annotate them as they were almost exclusively found in AC case. A previous study of Kennedy and Burgin considered these globules non-specific while later studies have suggested them to be glycoprotein inclusions^7,13^.

In conclusion, the mucin stainings PASD and ABPAS can be useful in the clinical setting, while mucicarmine is limited by a low sensitivity. Still, morphology and TTF-1 and p40 is sufficient for diagnosis in the majority of non-small cell lung cancer cases.
